# Prevalence and molecular characteristics of drug-resistant *Mycobacterium tuberculosis* in Beijing, China: 2006 versus 2012

**DOI:** 10.1186/s12866-016-0699-2

**Published:** 2016-05-12

**Authors:** Qing-Qin Yin, Wei-Wei Jiao, Qin-Jing Li, Fang Xu, Jie-Qiong Li, Lin Sun, Ying-Jia Li, Hai-Rong Huang, A-Dong Shen

**Affiliations:** Key Laboratory of Major Diseases in Children, Ministry of Education, National Key Discipline of Pediatrics (Capital Medical University), Beijing Key Laboratory of Pediatric Respiratory Infection Diseases, Beijing Pediatric Research Institute, Beijing Children’s Hospital, Capital Medical University, Beijing, China; National Clinical Laboratory on Tuberculosis, Beijing Key laboratory for Drug-resistant Tuberculosis Research, Beijing Chest Hospital, Capital Medical University, Beijing Tuberculosis and Thoracic Tumor Institute, Beijing, China

**Keywords:** Tuberculosis, Drug-resistant, Molecular characteristics

## Abstract

**Background:**

As the epidemic of MDR-TB and XDR-TB becomes increasingly severe, it is important to determine the clinical characteristics and molecular epidemiology of MDR-TB and XDR-TB. Recently, many studies have shown that clinical features and molecular characteristics of drug-resistant strains vary in different geographical areas, however, further information is needed to assess the dynamic evolution of drug-resistant TB. Comparative studies between different time periods are necessary to elucidate the development of drug-resistant TB.

**Results:**

A total of 255 and 537 strains were collected from Beijing Chest Hospital in 2006 and in 2012, respectively. Drug-resistance rates and mutations associated with resistance to first-line anti-tuberculosis (TB) drugs were compared. The overall rate of drug resistance among strains of TB in 2012 was 54.4 %, significantly higher than that in 2006 (34.9 %, *P* < 0.001). Rates of resistance to each first-line drug (isoniazid, rifampicin, streptomycin and ethambutol) and to second-line drug ofloxacin increased significantly from 2006 to 2012. The overall MDR rate also increased significantly from 2006 (14.9 %) to 2012 (27.0 %). The rate of MDR increased significantly between these two time periods in previously treated cases (*P* = 0.023) but not in new cases (*P* = 0.073), and the rate of XDR was similar in new cases at the two time periods, but was marginally higher in 2012 in previously treated cases (*P* = 0.056). Previous treatment was found to be a risk factor for drug-resistant TB, especially for MDR-TB. In addition, the proportion of drug resistant isolates in which *katG*, the *mabA-inhA* promoter, *oxyR-ahpC* intergenic region, *rpoB*, *rpsL*, and *embB* were mutated was similar in 2006 and 2012, however patterns of mutation in these loci were more diverse in 2012 compared to 2006.

**Conclusions:**

Our data suggests that the prevalence of drug resistant TB remains high in Beijing, China, and that increasing rates of resistance in *M. tuberculosis* to all anti-TB drugs should be considered when choosing an optimal anti-TB regimen. Moreover, acquired multi-drug resistance may play a primary role in the MDR-TB epidemic in Beijing, China. Consequently, this highlights the importance of an earlier start to effective and supervised treatment in order to reduce the burden of retreatment.

## Background

Although the incidence and mortality of tuberculosis (TB) have declined over the past decade, there were an estimated 9.0 million incident cases of TB and 1.5 million deaths in 2013 according to the World Health Organization [1]. Two major factors, including the lethal association of HIV with active TB disease, and the worldwide dissemination of multidrug-resistant (MDR) and extremely drug-resistant (XDR) strains of *Mycobacterium tuberculosis*, contribute to the severe TB epidemic [1]. Globally, an estimated 3.5 % of new cases and 20.5 % of previously treated cases have MDR-TB. In 2013 there were an estimated 480, 000 new cases of MDR-TB, and about 210, 000 associated deaths [1]. China is one of the 27 MDR-TB high-burden countries. According to the 2007 national survey of drug-resistant TB in China, 5.7 % of new cases and 25.6 % of previously treated cases were diagnosed with MDR-TB. Based on these survey results, it is estimated that there are 120, 000 new cases of MDR-TB in China per year [2]. In addition, XDR-TB cases were reported by over 100 countries in 2013, and up to 9.0 % of MDR-TB cases had XDR-TB in 2013 [1]. It is therefore urgent to control the epidemic of MDR-TB and XDR-TB.

In order to understand how drug-resistant TB develops and to find better ways to control MDR-TB and XDR-TB, it is essential to determine the clinical characteristics and molecular epidemiology of MDR-TB and XDR-TB. Recently, many studies have shown that clinical features and molecular characteristics of drug-resistant strains vary in different geographical areas [3, 4], such as, for example in different provinces in China [5–9]. These studies, however, have focused on the clinical and molecular characteristics of drug-resistant *M. tuberculosis* strains from a single time period, and are thus unable to assess the dynamic evolution of drug-resistant TB. Comparative studies between different time periods are thus necessary to elucidate the development of drug-resistant TB.

To better understand changes in the clinical and molecular characteristics of *M. tuberculosis* isolates in Beijing, China, we analyzed all strains collected from TB inpatients admitted to Beijing Chest Hospital (tertiary TB referral hospital) over two time periods separated by six years (2006 and 2012). Clinical information, drug-resistant phenotypes (including MDR and XDR phenotypes) and first-line drug resistance-associated mutations were compared between these two time periods.

## Results

### Demographic and clinical characteristics of enrolled subjects

Our purpose in this study was to evaluate changes in the clinical characteristics and molecular epidemiology of drug-resistant TB and thus assess its dynamic evolution. We thus performed a comparative study of *M. tuberculosis* isolates collected at the Beijing Chest Hospital over two time periods separated by 6 years. The MTB strain bank at Beijing Chest Hospital was first established in 2005, and complete datasets were available for each year from 2006 to 2012 when we initiated this project. We reasoned that a 6-year window would reveal possible changes in the rate of occurrence of drug-resistant TB, so chose to analyze all isolates collected from inpatients in the Beijing Chest Hospital from 2006 to 2012. A total of 792 isolates were selected, including 255 and 537 isolates from 2006 to 2012, respectively. The mean age of patients in 2006 and 2012 was 48.8 ± 19.5 years and 51.4 ± 19.6 years, and the male to female ratio was 2.7 and 2.9, respectively.

As shown in Table 1, there were no significant differences in the clinical characteristics (including gender, age and treatment history) of each subgroup (any drug-resistant TB, MDR-TB and pan-susceptible TB cases) between cases in 2006 and 2012 (*P* > 0.05). Of note, however, the proportion of previously treated cases in 2012 (40.8 %) was significantly higher than that in 2006 (31.0 %, *P* = 0.008).

**Table 1:** General demographic characteristics of patients enrolled in 2006 and 2012

Characteristics	Total			Any drug resistant TB			MDR-TB			Pan-susceptible TB		
	2006 (*n* = 255)	2012 (*n* = 537)	*P* value	2006 (*n* = 89)	2012 (*n* = 292)	*P* value	2006 (*n* = 38)	2012 (*n* = 145)	*P* value	2006 (*n* = 166)	2012 (*n* = 245)	*P* value
Gender												
Male	186 (72.9)	400 (74.5)	0.643	66 (74.2)	223 (76.4)	0.669	29 (76.3)	102 (70.3)	0.468	120 (72.3)	177 (72.2)	0.990
Female	69 (27.1)	137 (25.5)		23 (25.8)	69 (23.6)		9 (23.7)	43 (29.7)		46 (27.7)	68 (27.8)	
Age group (years)												
~25	41 (16.1)	76 (14.2)	0.682	14 (15.7)	46 (15.8)	0.728	6 (15.8)	32 (22.1)	0.428	27 (16.4)	30 (11.9)	0.271
~44	60 (23.5)	125 (23.3)		23 (25.8)	82 (28.1)		11 (28.9)	53 (36.6)		37 (20.9)	43 (17.5)	
~64	92 (36.1)	185 (34.5)		38 (42.7)	107 (36.6)		18 (47.4)	48 (33.1)		54 (32.2)	78 (32.5)	
>64	62 (24.3)	151 (28.1)		14 (15.7)	57 (19.5)		3 (7.9)	12 (8.3)		48 (30.5)	94 (38.1)	
Treatment history												
New cases	176 (69.0)	318 (59.2)	0.008	37 (41.6)	115 (39.4)	0.712	5 (13.2)	21 (14.5)	0.835	139 (83.7)	203 (82.9)	0.815
Previously treated cases	79 (31.0)	219 (40.8)		52 (58.4)	177 (60.6)		33 (86.8)	124 (85.5)		27 (16.3)	42 (17.1)	

### Drug-resistance patterns differ in 2006 and 2012

The overall rate of resistance in the MTB strains examined to any drug was 54.4 % in 2012, significantly higher than that in 2006 (34.9 %, *P* < 0.001), indicating the severe and worsening situation of drug-resistance in TB in China. Furthermore, the rate of resistance to any drug in 2012 was higher than that in 2006 in both new and previously treated cases (*P* < 0.05).

The proportion of drug-resistant *M. tuberculosis* isolates in 2006 was compared with that in 2012 (Table 2). The overall resistance level to each drug was significantly higher in 2012 (*P* < 0.05). Rates of resistance to rifampicin (RIF), streptomycin (STR), ethambutol (EMB) and ofloxacin (OFX) were significantly higher in new cases in 2012 (*P* < 0.05), while the proportion of isolates resistant to isoniazid (INH) were marginally higher (*P* = 0.054), and resistance to capreomycin (CAP) and amikacin (AMK) was not significantly changed (*P* > 0.05). The proportions of isolates resistant to INH, RIF, OFX and CAP in previously treated cases were significantly higher in 2012 (*P* < 0.05), but the proportions of isolates resistant to STR, EMB and AMK were not significantly changed (*P* > 0.05).

**Table 2:** Comparison of drug susceptibility patterns between clinical *M. tuberculosis* isolates in 2006 and in 2012

Susceptibility or resistance category	Total cases			New cases			Previously treated cases		
	2006 (*n* = 255) n (%)	2012 (*n* = 537) n (%)	*P* value	2006 (*n* = 176) n (%)	2012 (*n* = 318) n (%)	*P* value	2006 (*n* = 79) n (%)	2012 (*n* = 219) n (%)	*P* value
Any drug-resistance	89 (34.9)	292 (54.4)	<0.001	37 (21.0)	115 (36.2)	0.001	52 (65.8)	177 (80.8)	0.007
All first-line drug resistance	78 (30.6)	269 (50.1)	<0.001	30 (17.0)	98 (30.8)	0.001	48 (60.8)	171 (78.1)	0.003
INH	60 (23.5)	209 (38.9)	<0.001	20 (11.4)	57 (17.9)	0.054	40 (50.6)	152 (69.4)	0.003
RIF	43 (16.9)	164 (30.5)	<0.001	5 (2.8)	30 (9.4)	0.006	38 (48.1)	134 (61.2)	0.044
STR	59 (23.1)	189 (35.2)	0.001	21 (11.9)	63 (19.8)	0.026	38 (48.1)	126 (57.5)	0.149
EMB	24 (9.4)	88 (16.4)	0.008	8 (4.5)	31 (9.7)	0.040	16 (20.3)	57 (26.0)	0.306
All MDR	38 (14.9)	145 (27.0)	<0.001	5 (2.8)	21 (6.6)	0.073	33 (41.8)	124 (56.6)	0.023
INH + RIF	4 (2.0)	25 (4.5)	0.079	1 (0.6)	4 (1.3)	0.463	3 (3.8)	21 (9.6)	0.105
INH + RIF + STR/EMB	34 (13.3)	120 (22.3)	0.003	4 (2.3)	17 (5.3)	0.105	30 (38.0)	103 (47.0)	0.165
All second-line drug resistance	40 (15.7)	172 (32.0)	<0.001	14 (8.0)	45 (14.2)	0.042	26 (32.9)	127 (58.0)	<0.001
OFX	37 (14.5)	164 (30.5)	<0.001	13 (7.4)	42 (13.2)	0.049	24 (30.4)	122 (55.7)	<0.001
CAP	1 (0.4)	29 (5.4)	0.001	1 (0.6)	2 (0.6)	0.934	0	27 (12.3)	0.001
AMK	9 (3.5)	42 (7.8)	0.021	1 (0.6)	7 (2.2)	0.168	8 (10.1)	35 (16.0)	0.204
All pre-XDR	17 (6.7)	73 (13.6)	0.004	2 (1.1)	10 (3.1)	0.165	15 (19.0)	63 (28.8)	0.090
All XDR	6 (2.4)	36 (6.9)	0.008	1 (0.6)	4 (1.3)	0.463	5 (6.3)	32 (14.6)	0.056

The overall rate of MDR-TB was 27.0 % in 2012, significantly higher than that in 2006 (14.9 %, *P* < 0.05). The proportion of MDR-TB in previously treated cases was significantly higher in 2012 compared to 2006 (*P* = 0.023), but there was no significant increase in new cases (*P* = 0.073). The overall pre-XDR rate was 13.6 % in 2012, again, significantly higher than that in 2006 (6.7 %, *P* = 0.004). However, the pre-XDR rate in 2012 did not increase significantly in either new cases (*P* = 0.165) or previously treated cases (*P* = 0.090) compared with 2006. The rate of XDR-TB also increased significantly from 2006 (2.4 %) to 2012 (6.9 %). While the rate of XDR was similar in new cases during the two time periods, it was marginally higher in 2012 in previously treated cases (*P* = 0.056).

### Factors associated with drug-resistant TB and MDR-TB

The risk factors associated with resistance to any drug and MDR-TB were analyzed based on pooled demographic data for all patients (Table 3). By univariate analysis, age and treatment history were significantly associated with resistance to any drug and MDR-TB (*P* < 0.05). Multivariate analysis confirmed that age and treatment history were independently associated with resistance to any drug and MDR-TB (*P* < 0.05).

**Table 3:** Factors associated with drug-resistant tuberculosis in all patients

Factors	Resistance to any drug (*n* = 381)	MDR-TB (*n* = 183)	Pan-susceptible TB (*n* = 411)	MDR-TB vs Pan-susceptible TB				Resistance to any drug vs Pan-susceptible TB			
				Odds ratio (95 % CI)	*P* value	Adjusted odds ratio (95 % CI)	*P* value	Odds ratio (95 % CI)	*P* value	Adjusted odds ratio (95 % CI)	*P* value
Gender											
Male	289	131	297	Reference				Reference			
Female	92	52	114	1.03 (0.70–1.52)	0.865			0.83 (0.60–1.14)	0.250		
Age group (years)											
~25	60	38	57	Reference		Reference		Reference		Reference	
~44	105	64	80	1.20 (0.71–2.03)	0.892	1.05 (0.51–2.19)	0.718	1.25 (0.78–1.99)	0.734	1.09 (0.65–1.83)	0.625
~64	145	66	132	0.75 (0.45–1.24)	0.155	0.60 (0.30–1.21)	0.033	1.04 (0.68–1.61)	0.969	0.99 (0.61–1.60)	0.610
>64	71	15	142	0.16 (0.08–0.31)	<0.001	0.12 (0.05–0.27)	<0.001	0.48 (0.30–0.75)	0.011	0.52 (0.31–0.86)	0.002
Treatment history											
New cases	152	26	342	Reference		Reference		Reference		Reference	
Previously treated cases	229	157	69	29.93 (18.35–48.81)	<0.001	32.64 (19.40–54.92)	<0.001	7.47 (5.37–10.39)	<0.001	7.11 (5.09–9.92)	<0.001

Patients older than 64 years had a significantly lower risk of developing drug resistance relative to patients in the younger age group (<25 years), with an adjusted OR of 0.52 (95 % CI: 0.31–0.86, *P* = 0.002). This risk decreased significantly in MDR-TB cases, with an adjusted OR of 0.12 (95 % CI: 0.05–0.27, *P* < 0.001). Previously treated cases were associated with a higher risk of developing drug resistance, with an adjusted OR of 7.11 (95 % CI: 5.09–9.92, *P* < 0.001), and the risk increased significantly in MDR-TB cases, with an adjusted OR of 32.64 (95 % CI: 19.40–54.92, *P* < 0.001).

### Drug resistance associated mutations in 2006 and 2012

Using a combination of mutations in *katG*, the *mabA-inhA* promoter and the *oxyR-ahpC* intergenic region, DNA sequencing was able to identify 86.7 % (52/60) and 85.6 % (179/209) of INH-resistant isolates collected in 2006 and 2012, respectively. Similarly, 88.4 % (38/43) and 92.1 % (151/164) of RIF-resistant isolates were detected based on mutations in the RRDR region of *rpoB*, 79.7 % (47/59) and 76.2 % (144/189) of STR-resistant isolates were detected based on mutations in *rpsL*, and 54.2 % (13/24) and 62.5 % (55/88) of EMB-resistant isolates were detected based on mutations in *embB*. In addition, 8.2 % (19/231) and 11.4 % (51/449) of EMB-susceptible isolates in the 2006 and 2012 groups were also found to harbor mutations in the *embB* gene. Other target mutations were not found in INH, RIF and STR susceptible isolates in the 2006 and 2012 collections.

The frequencies of common mutations in INH, RIF, STR or EMB resistant isolates were similar in 2006 and in 2012: *katG*315 (71.7 % vs 58.4 %, *P* = 0.062), *mabA-inhA −*15 (15.0 % vs 19.6 %, *P* = 0.418); *rpoB*531 (55.8 % vs 63.4 %, *P* = 0.361), *rpoB*526 (16.3 % vs 17.1 %, *P* = 0.902); *rpsL*43 (64.4 % vs 64.0 %, *P* = 0.975); *embB*306 (33.3 % vs 35.2 %, *P* = 0.863). Data on common mutation patterns in these drug-resistant isolates are shown in Table 4. In addition, the *embB306* mutation was detected in 4.5 % (11/231) and 6.5 % (29/449) of EMB-susceptible isolates in the 2006 and 2012 collections, respectively. Moreover, patterns of mutation in the loci examined were more diverse in 2012 compared to 2006.

**Table 4:** Most frequently identified mutations within first-line drug-resistance associated loci among drug-resistant *M. tuberculosis* isolates

Drug	Locus	Mutated position	2006		2012		Total	
			Mutated patterns	Relative frequency % (No. of mutant isolates/No. of drug-resistant isolates)	Mutated patterns	Relative frequency % (No. of mutant isolates/No. of drug-resistant isolates)	Mutated patterns	Relative frequency % (No. of mutant isolates/No. of drug-resistant isolates)
INH	*katG*	Codon 315	AGC→ACC	68.3 (41/60)	AGC→ACC	56.5 (118/209)	AGC→ACC	59.1 (159/269)
			AGC→ACA	1.7 (1/60)	AGC→ACA	0.5 (1/209)	AGC→ACA	0.7 (2/269)
			AGC→AAC	1.7 (1/60)	AGC→AAC	1.0 (2/209)	AGC→AAC	1.1 (3/269)
					AGC→CGC	0.5 (1/209)	AGC→CGC	0.3 (1/269)
		Other mutations	–	0	–	7.7 (16/209)	–	6.0 (16/269)
	*mabA-inhA* promoter	−15	−15 C→T	15.0 (9/60)	−15 C→T	19.6 (41/209)	−15 C→T	18.6 (50/269)
		Other mutations	–	6.7 (4/60)	–	3.3 (7/209)	–	4.1 (11/269)
	*oxyR-ahpC* intergenic region	−10	−10 C→T	0	−10 C→T	2.4 (5/209)	−10 C→T	1.9 (5/269)
		−30	−30 C→T	1.7 (1/60)	−30 C→T	0	−30 C→T	0.4 (1/269)
		−39	−39 C→T	0	−39 C→T	2.4 (5/209)	−39 C→T	1.9 (5/269)
		Other mutations	–	1.7 (1/60)	–	3.8 (8/209)	–	3.3 (9/269)
RIF	*rpoB* RRDR	Codon 531	TCG→TTG	53.5 (23/43)	TCG→TTG	60.4 (99/164)	TCG→TTG	58.9 (122/207)
			TCG→TGG	2.3 (1/43)	TCG→TGG	1.2 (2/164)	TCG→TGG	1.4 (3/207)
					TCG→TTC	1.2 (2/164)	TCG→TTC	1.0 (2/207)
					TCG→CAG	0.6 (1/164)	TCG→CAG	0.5 (1/207)
		Codon 526	CAC→TAC	9.3 (4/43)	CAC→TAC	7.3 (12/164)	CAC→TAC	7.7 (16/207)
			CAC→GAC	4.7 (2/43)	CAC→GAC	2.4 (4/164)	CAC→GAC	2.9 (6/207)
			CAC→AAC	2.3 (1/43)	CAC→AAC	1.2 (2/164)	CAC→AAC	1.4 (3/207)
					CAC→CTC	4.3 (7/164)	CAC→CTC	3.4 (7/207)
					CAC→ACC	0.6 (1/164)	CAC→ACC	0.5 (1/207)
					CAC→GTC	0.6 (1/164)	CAC→GTC	0.5 (1/207)
					CAC→TGC	0.6 (1/164)	CAC→TGC	0.5 (1/207)
		Other mutations	–	16.3 (7/43)	–	11.6 (19/164)	–	12.6 (26/207)
STR	*rpsL*	Codon 43	AAG→AGG	59.3 (35/59)	AAG→AGG	63.5 (120/189)	AAG→AGG	62.5 (155/248)
			AAG→AAC	5.1 (3/59)	AAG→ACG	0.5 (1/189)	AAG→AAC	1.2 (3/248)
							AAG→ACG	0.4 (1/248)
		Other mutations	–	15.3 (9/59)	–	12.2 (23/189)	–	12.9 (32/248)
EMB	*embB*	Codon 306	ATG→GTG	25.0 (6/24)	ATG→GTG	19.3 (17/88)	ATG→GTG	29.5 (23/112)
			ATG→ATA	8.3 (2/24)	ATG→ATA	11.4 (10/88)	ATG→ATA	10.7 (12/112)
					ATG→ATT	2.3 (2/88)	ATG→ATT	1.8 (2/112)
					ATG→ATC	1.1 (1/88)	ATG→ATC	0.9 (1/112)
					ATG→CTG	1.1 (1/88)	ATG→CTG	0.9 (1/112)
		Other mutations	–	20.8 (5/24)	–	27.3 (24/88)	–	25.9 (29/112)

## Discussion

In this hospital-based study, the rate of resistance to any drug and the MDR rate of *M. tuberculosis* isolates was found to increase significantly from 2006 to 2012, reflecting the serious drug-resistant TB epidemic in China. Overall, the percentage of previously treated cases in 2012 was higher than that in 2006, suggesting that treatment of previously treated cases is still a big challenge in controlling the TB epidemic in China. Furthermore, the proportion of previously treated cases among the MDR-TB cases increased to 40.8 % in 2012 compared to that in 2006 (31.0 %). This implies that acquired multi-drug resistance may play an increasing role in the MDR-TB epidemic in China.

Among the new TB cases in this study, rates of resistance to each first-line drug in the 2006 isolate collection were consistent with those found in the National survey of drug-resistant TB conducted in 2007 [2]. However, rates of resistance to each first-line drug were significantly increased in 2012. The rate of resistance to the second-line drug OFX also increased from 7.4 % in 2006 to 13.2 % in 2012 among new TB cases. The increasing rate of OFX resistance rate should thus be considered when choosing an optimal anti-TB regimen. As the new cases in this study had not received therapy or were in treatment for less than 1 month, the rate of drug resistance among new cases should reflect the transmission of drug-resistant TB.

Among previously treated cases examined in our study, rates of resistance of *M. tuberculosis* isolates to INH, RIF, OFX and CAP were also higher in the 2012 collection compared with the 2006 collection. Overall rates of drug-resistance to each first-line drug in 2006 were much higher than that in the National survey [2]. This difference might be attributed to differences in sampling methods and the subjects targeted. For the national survey, isolates were selected from across 10 provinces of China using a cluster-randomized sampling method [2]. In contrast, in this study, patients were recruited from one tertiary TB referral hospital, i.e., most subjects were hospitalized TB patients with relatively serious symptoms. Overall, the increasing rate of drug-resistance in previously treated cases underlines the importance of standard treatment and the necessity of optimizing the treatment regimen according to the results of drug susceptibility testing.

The MDR and XDR rates in the 2006 isolates were similar to those found in the National survey [2] among new cases, but were much higher for previously treated cases. This may also be due to differences in the patients targeted as discussed above. Moreover, the rates of MDR and XDR MTB isolates had increased in 2012 compared to 2006 both among new cases and previously treated cases, although the difference was not significant for new cases. This data suggests that acquired MDR might outweigh primary resistance in the MDR epidemic in China. This finding is different from that of Gao et al. [10] who found that it is recent transmission of *M. tuberculosis* including transmission of MDR strains, that contributes most highly to the TB epidemic in China. This difference in findings may be due to variation in regions sampled: Gao et al. collected strains from five counties within five different provinces [10], while the study population examined here came from one hospital in Beijing. Previous studies have shown that drug-resistance rates vary in different provinces [11]. Pre-XDR rates increased significantly from 2006 to 2012. Since pre-XDR is an important step in the development of drug resistance from MDR to XDR, this finding presents an additional alarming indication of the worsening situation of XDR-TB in China.

Previous treatment is a well-known risk factor for drug-resistant TB and MDR-TB, and the prevalence of MDR-TB can be up to 10 times higher after unsuccessful treatment [12]. In this sense, results obtained here are consistent with previous observations: the risk of suffering MDR-TB among previously treated cases was 32.64 times higher than that in new cases. The implementation of DOTS is thus still very important for effectively controlling drug-resistant TB and MDR-TB, especially with respect to supervising patients to complete the treatment. We also found that people older than 64 years of age had a lower risk of drug-resistant TB and MDR-TB. This is consistent with the conclusion of a systematic review of European studies which concluded that MDR-TB cases are more likely to occur in patients younger than 65 years of age [13]. The higher risk of getting MDR-TB in people under 65 years may be attributed to the use of RIF for anti-TB treatment from around 1965. TB cases in older patients are usually considered as relapse cases, and the infecting strains may be more ancient, and carry a lower risk of becoming resistant to RIF.

Between 2006 and 2012, there was more or less no difference in the molecular detection rate for first line drug-resistance in our study. It should be noted that mutations in *embB* were also found in EMB-susceptible isolates [14]. Here, *embB*306 mutations were found in 4.5 % and 6.5 % of EMB-susceptible isolates in 2006 and 2012, respectively. This percentage is lower than previously reported in Russia (31.2 %) [14] and Singapore (20.0 %) [15], and may be due to different percentages of multi-drug resistance among EMB-susceptible isolates. Hazbon et al. collected 807 *M. tuberculosis* isolates from Colombia, Mexico, New York and Texas, and found that the association between *embB306* mutations and resistance to increasing numbers of anti-TB drugs was significant in each region, suggesting the role of *embB306* mutations in broad drug resistance [16]. The frequency of the *embB306* mutation in one setting may thus be influenced by the percentage of multi-drug resistant isolates. Accordingly, the *embB306* mutation does not appear to be a reliable marker for predicting EMB resistance in Beijing, China.

Mutation rates at common loci in specific genes associated with drug-resistance were similar in 2006 and 2012. It has previously been shown that antibiotic resistance associated mutations can impair bacterial fitness [17, 18]. However, acquisition of compensatory mutations in drug resistant strains can restore their ability to survive. It is possible that the reason why the mutation rate at common loci in the drug-resistant isolates in our study was unchanged may be that mutations in these common loci are associated with other compensatory mutations that lead to lower fitness costs.

Our study, however, has some limitations. The isolates examined were collected from only one TB referral hospital in Beijing. Patients admitted to this hospital tend to be severe cases or to have received therapy in other hospitals but with poor effect. Thus the incidence of drug-resistant TB may be overestimated, and may not reflect the average level of the whole country. Well-designed studies with a wide coverage of different regions in China should thus be conducted in the future.

## Conclusions

Results from this study indicate that the prevalence of drug resistant TB remains high in Beijing, China, and suggest that increasing rates of resistance in *M. tuberculosis* to all anti-TB drugs should be considered when choosing optimal anti-TB regimens for treatment. The rate of MDR and XDR in *M. tuberculosis* isolates was higher in 2012 compared to 2006, especially in isolates from previously-treated cases, suggesting that acquired multi-drug resistance may increasingly be playing a primary role in the MDR-TB epidemic in China. These findings highlight the importance of an earlier start on effective and supervised treatment in order to reduce the burden of retreatment.

## Methods

### *M. tuberculosis* isolates and drug susceptibility testing (DST)

A total of 255 and 537 *M. tuberculosis* isolates collected in 2006 and 2012, respectively, were obtained from Beijing Bio-Bank of clinical resources on Tuberculosis at Beijing Chest Hospital. All isolates were recovered from inpatients diagnosed with pulmonary TB. If several isolates had been recovered from the same patient at different time points, only the earliest isolate was included in this analysis. Clinical investigations were conducted in accordance with the principles expressed in the Declaration of Helsinki, and this study was approved by the Ethics Committee of Beijing Chest Hospital. Written informed consent was not obtained from patients as the data were analyzed anonymously.

DST was performed using the proportion method on Löwenstein-Jensen medium, according to WHO guidelines, with the following concentrations of anti-TB drugs: INH - 0.2 μg/ml, RIF - 50 μg/ml, STR - 10 μg/ml, EMB - 5.0 μg/ml, OFX - 2.0 μg/ml, levofloxacin (LFX) - 2.0 μg/ml, CAP - 40 μg/ml, AMK - 30 μg/ml. Strains were deemed to be resistant to a specific drug when the growth rate was ≥1 % that of the control. Both OFX and LFX susceptibility testing were performed, but as results showed that all LFX-resistant isolates were also resistant to OFX, LFX-resistance data were not included in the analysis.

### Data collection and definitions

Demographic and clinical information on enrolled patients, including gender, age, address and TB treatment history, were obtained from inpatients’ medical records. New cases were TB patients who had never been treated with anti-TB drugs or that had been treated for less than 1 month. Previously treated cases were TB patients who had been treated with anti-TB drugs for 1 month or longer. MDR-TB was defined as resistance to at least INH and RIF [19]. Although kanamycin resistance is included in the WHO definition of pre-XDR and XDR, this drug is rarely used to treat TB at the Beijing Chest Hospital. In this study, XDR-TB was therefore defined as resistance to INH and RIF plus OFX and at least one injectable second-line drug (CAP or AMK). Pre-XDR TB was defined as resistance to INH and RIF plus either OFX or a second-line injectable drug (CAP or AMK), but not both.

### Detection of drug resistance associated gene mutations

Loci associated with hot-spots of drug-resistance to first line anti-TB drugs (INH, RIF, STR and EMB), including *katG*, the *mabA-inhA* promoter, *oxyR-ahpC* intergenic region, *rpoB* RRDR (RIF-resistance-determining region), *rpsL* and *embB* were sequenced in this study. Genomic DNA was extracted from freshly cultured *M. tuberculosis* using a conventional cetyltrimethylammonium bromide (CTAB) method [20]. All the primers for amplification of target nucleotide positions and DNA sequencing are listed in Table 5. For each target gene, the volume of PCR mixture was 25 μL, containing 12 μL of 2× Taq Master Mix, 1 μL of forward and reverse primers (10 μΜ), 10 μL of distilled H_2_O and 1 μL of genomic DNA. For *katG, rpoB* RRDR*, embB,* and *rpsL*, the PCR program comprised an initial denaturation at 95 °C for 3 min, followed by 35 cycles of 94 °C for 45 s, 62 °C for 45 s and 72 °C for 35 s, and a final step of 72 °C for 4 min. For the *mabA-inhA* promoter and *oxyR-ahpC* intergenic region, the PCR program comprised an initial denaturation at 95 °C for 5 min, followed by 35 cycles of 94 °C for 1 min, 62 °C for 1 min and 72 °C for 1 min, and a final step of 72 °C for 4 min. All PCR products were sent for sequencing using primers which were the same as those used for PCR amplification. Sequencing data was aligned with the corresponding sequences of the *M. tuberculosis* H37Rv reference strain using BLASTn optimized for megablast on the National Center for Biotechnology Information website (http://blast.ncbi.nlm.nih.gov/Blast.cgi).

**Table 5:** Primers for PCR amplification and DNA sequencing

Gene	Amplified Region (bp)	Orientation	Oligonucleotide sequence (5′-->3′)	Tm (°C)
*katG*	580 to 1257	Forward	GATGAGGTCTATTGGGGCAAG	59.8
		Reverse	GTCTCGGTGGATCAGCTTGTA	59.8
*mabA-inhA* promoter	−436 to 182 (*mabA*)	Forward	ATGCGCTCTTCCCAGACTT	58.0
		Reverse	TCACATTCGACGCCAAACAG	60.0
*OxyR* ^*’*^ *-ahpC* intergenic region	285 (*oxyR* ^*’*^) to 312 (*ahpC*)	Forward	CCCTCATGCAGTCACAACAA	60.0
		Reverse	TTTGAGGTCGTTGTGCTGTG	60.0
*rpoB* RRDR	916 to 1572	Forward	GGTCGCTATAAGGTCAACAAGAAG	61.0
		Reverse	GTACACGATCTCGTCGCTAACC	62.1
*embB*	847 to 1581	Forward	GTGATATTCGGCTTCCTGCTCT	60.2
		Reverse	GTAGTAGTAACGCAGGTTCTCGGTA	62.9
*rpsL*	254 to 877	Forward	GAATCGAGTTTGAGGCAAGCTAT	58.8
		Reverse	CTCAAGCGCACCATAAACAAT	55.9

### Statistical analysis

Pearson chi-square tests or Fisher exact tests were used to compare drug-resistance rates between isolates collected in 2006 and in 2012. Univariate analysis of categorical variables was performed with the Pearson chi-square test or Fisher exact test as appropriate. Univariate and multivariate logistic regression analyses were used to analyze drug-resistance-associated risk factors. Variables with a *P* value less than 0.05 in the univariate analysis were analysed further by multivariable logistic regression analysis. A two-sided *P* value of <0.05 was considered statistically significant. Statistical analyses were performed using SPSS statistics 19.0.

## Ethics approval and consent to participate

This study was approved by the Ethics Committee of Beijing Chest Hospital. Written informed consent was not obtained from patients as the data were analyzed anonymously.

## Availability of data and materials

All of the data are complete in this study, no supplementary data are attached.

## Abbreviations

AMK: amikacin
CAP: capreomycin
CTAB: cetyltrimethylammonium bromide
DST: drug susceptibility testing
EMB: ethambutol
INH: isoniazid
LFX: levofloxacin
MDR: multidrug-resistant
OFX: ofloxacin
RIF: rifampicin
RRDR: rifampicin-resistance-determining region
STR: streptomycin
TB: tuberculosis
XDR: extremely drug-resistant
